# Effect of Erythropoiesis-Stimulating Agent Types on Hemoglobin Variability in Hemodialysis Patients

**DOI:** 10.3390/jcm14144863

**Published:** 2025-07-09

**Authors:** Seok-Hui Kang, A-Young Kim

**Affiliations:** Division of Nephrology, Department of Internal Medicine, College of Medicine, Yeungnam University, Daegu 42415, Republic of Korea; kangkang@ynu.ac.kr

**Keywords:** erythropoiesis-stimulating agent, hemodialysis, hemoglobin variability

## Abstract

**Background:** Our study aimed to evaluate the differences in hemoglobin variability among patients undergoing hemodialysis (HD) treated with three types of erythropoiesis-stimulating agents (ESAs) and the association between hemoglobin variability and clinical outcomes. **Methods:** In this study, data from the 6th and 7th HD quality assessments were used, comprising 48,726 patients in South Korea. ESAs are categorized into short-acting (epoetin alfa/beta/delta, requiring more frequent administration), intermediate-acting (darbepoetin alfa), and long-acting agents (methoxy polyethylene glycol-epoetin beta, requiring extended dosing intervals), each with distinct pharmacokinetic properties and dosing schedules. Based on use of ESA types, participants were divided into the following groups: Short, Intermediate, and Long. Hemoglobin levels were measured monthly over a 6-month assessment period. Hemoglobin variability was defined as the residual standard deviation derived from a within-subject linear regression model with six hemoglobin values for each patient. **Results:** The Short, Intermediate, and Long groups comprised 36,420, 10,514, and 1792 patients, respectively. The hemoglobin variability (mean [95% confidence interval]) was 0.60 (0.60–0.60), 0.68 (0.67–0.68), and 0.64 (0.62–0.65) g/dL in the Short, Intermediate, and Long groups, respectively. Multivariate and subgroup analyses revealed that the hemoglobin variability was lower in the Short group than in the other two groups. Cox regression did not show a significant association between an increase in hemoglobin variability and all-cause mortality or cardiovascular events in univariate and multivariate analyses. **Conclusions:** Our cohort study found that the use of short-acting ESAs showed the lowest hemoglobin variability, whereas the use of intermediate-acting ESAs showed the highest variability. In the context of South Korea’s healthcare system, where frequent hemoglobin monitoring and strict ranges are emphasized, short-acting ESAs combined with regular laboratory follow-up appeared to support more stable hemoglobin levels.

## 1. Introduction

Anemia is prevalent among patients undergoing hemodialysis (HD) and is commonly managed with erythropoiesis-stimulating agents (ESAs). ESA therapy plays a critical role in maintaining target hemoglobin levels; however, fluctuations in hemoglobin levels have been linked to adverse clinical outcomes, including increased mortality [1,2]. An understanding of the factors contributing to hemoglobin variability, particularly the type of ESA prescribed, is crucial for optimizing anemia management strategies for patients undergoing HD.

ESAs are categorized into short-, intermediate-, and long-acting agents, each with distinct pharmacokinetics and dosing schedules. Short-acting agents, such as epoetin-alfa/beta/delta, require more frequent administration, whereas intermediate-acting agents (darbepoetin-alfa) and long-acting agents (methoxy polyethylene glycol-epoetin beta) allow for extended dosing intervals [3]. These pharmacological differences are thought to influence the degree of hemoglobin fluctuations. Hemoglobin variability is not merely a laboratory artifact; it is associated with tissue hypoxia, cardiovascular stress, and adverse clinical outcomes, including increased mortality. Therefore, minimizing hemoglobin variability represents a clinically relevant objective in the management of dialysis-related anemia.

Some studies suggest that long-acting ESAs reduce hemoglobin variability due to less frequent dosing and more stable erythropoiesis; however, others indicate no significant difference or even higher hemoglobin variability with specific agents [4,5,6,7,8,9]. Bernieh et al. performed a randomized controlled trial involving 139 patients undergoing HD in the United Arab Emirates and reported lower hemoglobin variability with intermediate-acting ESAs than short acting agents [5]. Another study involving patients undergoing peritoneal dialysis in the United Kingdom compared long-acting and short-acting ESAs, reporting a similar trend of lower hemoglobin variability with long-acting agents [9]. These findings suggest that hemoglobin variability decreases as the half-life of ESAs increases. However, a study conducted in Australia involving patients undergoing predialysis, peritoneal dialysis, and HD compared intermediate-acting and short-acting agents, showing greater hemoglobin variability with intermediate-acting agents [6]. A study conducted in Japan involving patients undergoing predialysis shows greater hemoglobin variability with long-acting ESAs than with intermediate-acting agents [7]. These findings indicate that hemoglobin variability increases as the half-life of ESAs increases. Similarly, data from a European population of patients with non-dialysis chronic kidney disease show a similar trend in hemoglobin variability with intermediate-acting and long-acting agents [8]. Consequently, findings across the existing literature remain inconsistent.

While long-acting agents are generally presumed to maintain more stable hemoglobin levels—due to fewer dose adjustments and less frequent injections than short-acting or intermediate-acting agents—existing data show inconsistent results. Beyond biological effects and individual drug responses, these discrepancies may reflect differences in study design, population characteristics, follow-up duration, and regional clinical practice guidelines. Furthermore, individual patient characteristics and responsiveness to ESAs remain critical factors in anemia management. Therefore, region-specific or nation-specific large-scale data are necessary to clarify the relationship between ESA type and hemoglobin variability in real-world clinical settings. In regions without strong recommendations for strict hemoglobin targets, hemoglobin variability may remain at elevated levels regardless of the ESA types. In clinical settings with high provider workload and fewer reimbursement restrictions on ESA prescription, long-acting ESAs may be preferred. In these settings, ESA agents may be administered continuously regardless of hemoglobin levels, potentially contributing to lower hemoglobin variability. Consequently, the choice of ESA and the resulting hemoglobin variability can be influenced by multiple real-world factors, including healthcare provider workload, clinical practice patterns, drug costs, reimbursement policies, and national guidelines for hemoglobin management. In healthcare systems such as that of South Korea, where ESA reimbursement is tightly regulated and strict hemoglobin targets are enforced, short-acting ESAs are often preferred. This may lead to a distinct pattern of hemoglobin variability associated with different ESA types. Investigating this association could provide valuable insights into optimizing anemia management and improving clinical outcomes in patients undergoing HD.

The unique healthcare system of South Korea, which emphasizes maintaining hemoglobin within a narrow target range through frequent laboratory monitoring and timely dose adjustments, may influence this relationship. Consequently, findings from this setting may offer meaningful insights for other regions with similar clinical practices, patient populations, or prescribing patterns. This study aims to address this gap by analyzing a large, nationally representative cohort of patients undergoing HD to evaluate differences in hemoglobin variability across ESA types—short-acting, intermediate-acting, and long-acting—and to examine the relationship between hemoglobin variability and clinical outcomes. This nationally representative approach provides meaningful evidence to inform ESA selection and support individualized anemia management in routine nephrology practice.

## 2. Materials and Methods

### 2.1. Data Sources and Study Population

**Figure 1:**
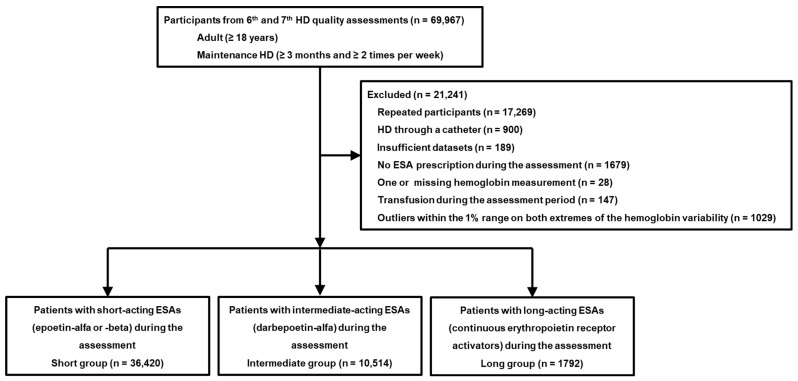
Study flowchart illustrating patient selection, inclusion and exclusion criteria, and stratification steps. Abbreviations: HD, hemodialysis; ESA, erythropoiesis-stimulating agent.

In our retrospective study, datasets from patients who underwent periodic HD quality assessment and their claims data were analyzed [10,11]. In brief, the first HD quality assessment program was performed between October and December 2010. Data from the sixth (March and August 2018) and seventh (October 2020 and March 2021) HD quality assessment programs were used in this study (Figure 1).

These programs included adult patients (≥18 years) who had undergone maintenance HD (≥3 months and ≥2 times per week). Data from the relevant HD quality assessment and claims of all patients were analyzed.

Among 69,967 patients included in the sixth and seventh assessments, we excluded the following participants: repeat participants (*n* = 17,269), those undergoing HD through a catheter (*n* = 900), those with insufficient data (*n* = 189), those without an ESA prescription during the assessment (*n* = 1679), those with at least one missing hemoglobin measurement during the assessment (*n* = 28), those identified as outliers within the 1% range on both extremes of the hemoglobin variability (*n* =1029), and those who received transfusions during the assessment period (*n* = 147). The exclusion of extreme outliers (top and bottom 1%) was intended to minimize the influence of non-representative or potentially spurious values on the analysis. These values may be due to measurement errors, transient clinical events (e.g., acute bleeding), or data entry inaccuracies, and are not reflective of the typical hemoglobin control or response to ESA therapy of the patient. These outliers can distort measures of central tendency and variance, potentially skewing the interpretation of variability. In the total cohort, hemoglobin variability was 0.58 ± 0.28 g/dL. However, the median (interquartile range) hemoglobin variability for the top and bottom 1% was 1.72 (1.63–1.88) g/dL and 0.10 (0.08–0.12) g/dL, respectively. These values deviate substantially from those of the overall cohort and could exert a disproportionate influence on the results. Overall, 48,726 patients were included in this study. This study was approved by the institutional review board of Yeungnam University Medical Center (approval no. YUMC 2023-12-012). Informed consent was not required as patient records and information were anonymized and de-identified before analysis. The institutional review board of Yeungnam University Medical Center waived the informed consent requirement owing to the retrospective nature of the study. This research was conducted in adherence to ethical standards outlined in the Declaration of Helsinki.

### 2.2. Study Variables

During each HD quality assessment, data were collected on age, sex, HD vintage (months), and vascular access type. Additional clinical measures included hemoglobin (g/dL), body mass index (kg/m^2^), Kt/V_urea_ (hemodialysis adequacy), serum albumin (g/dL), serum calcium (mg/dL), serum phosphorus (mg/dL), serum creatinine (mg/dL), and ultrafiltration volume (L/session). These data were collected monthly, with all laboratory values averaged from the monthly recordings. Kt/V_urea_ was calculated using the Daugirdas equation [12].

Hemoglobin levels were measured monthly over a 6-month assessment period. Hemoglobin variability was defined as the residual standard deviation derived from a within-subject linear regression model with six hemoglobin values for each patient, as described in a previous study [13]. The ESA dose (international unit (IU)/week) was averaged over the 6-month period. The erythropoietin resistance index (ERI) was calculated using the following equation: ERI = ESA dose (IU/week)/body weight (kg)/hemoglobin level (g/dL) [14].

We used medication codes from the Korea Health Insurance Review and Assessment, which have been described in a previous study [10]. Participants were divided into the following groups based on the ESA type used during the 6-month assessment period: Short, Intermediate, and Long. Patients using epoetin-alfa or epoetin-beta were placed in the Short group, and those using darbepoetin-alfa were placed in the Intermediate group. Patients using continuous erythropoietin receptor activators were classified into in Long group. Patients who used ≥2 ESAs were assigned to the ESA group corresponding to the highest doses taken over the 6 months. The doses of various ESAs were converted to a uniform unit (IU/week) using a conversion ratio from a previous study [15].

Medications including renin–angiotensin system blockers (RASBs), aspirin, clopidogrel, anti-hypertensive drugs, and statins were evaluated. Medication use was defined as ≥1 prescription identified during the HD quality assessment program. Comorbidities were assessed for 1 year before HD quality assessment. The Charlson Comorbidity Index (CCI) was used to define comorbidities, which include 17 comorbidities. CCI scores were calculated for all patients [16,17]. Additionally, myocardial infarction (MI) or congestive heart failure (CHF) was identified using ICD-10 codes.

Patients were followed up until June 2024. All-cause mortality and cardiovascular events (CVE) were analyzed. Data on patient death were obtained from the Health Insurance Review and Assessment Service, and patients who changed to peritoneal dialysis or received kidney transplantation without experiencing an event were censored at the time of transfer. The incidence of CVE including MI, stroke, and revascularization regardless of survival or death, was evaluated as previously described [18]. The incidence of CVE was evaluated using patients without CVE for 6 months during the HD quality assessment program and 1 year before the program.

### 2.3. Statistical Analyses

Data were analyzed using SAS Enterprise Guide v.7.1 and R v.3.5.1. Categorical variables are presented as frequencies and percentages, and continuous variables are expressed as means with standard deviations (or 95% confidence intervals (CIs)). The statistical significance between categorical variables was assessed using Pearson’s χ^2^ test or Fisher’s exact test. Differences between continuous variables were examined using one-way analysis of variance with Tukey’s post-hoc test.

Multivariate analyses were performed by analysis of covariance and adjusted for the following variables: age, sex, body mass index, vascular access type, diabetes, HD vintage, CCI score, ultrafiltration volume, Kt/V_urea_, levels of hemoglobin, serum albumin, serum creatinine, serum phosphorus, and serum calcium, use of RASBs, statins, clopidogrel, anti-hypertensive drugs, or aspirin, presence of MI or CHF, ESA dose, and ERI. Subgroup analyses were based on age, sex, median CCI (7 as median value), HD vintage (40 months as median value), and ESA dose (<6960 IU/week for the low group and ≥6960 IU/week for the high group). The statistical significance and association for each variable were analyzed by linear regression analysis. Hazard ratios (HRs) and CIs for mortality or CVE were calculated by Cox regression. Statistical significance was determined at *p* < 0.05.

## 3. Results

### 3.1. Baseline Characteristics

The Short, Intermediate, and Long groups comprised 36,420, 10,514, and 1792 patients, respectively (Table 1).

In comparison with patients in other groups, those in the Short group were younger and had higher HD vintage, ultrafiltration volume, serum albumin level, phosphorus level, calcium level, creatinine level, ESA dose, and ERI. In addition, compared with the other two groups, the Short group exhibited lower Kt/V_urea_, lower usage of RASBs and statins, and a lower prevalence of MI or CHF. There were lower proportions of males and patients using aspirin or clopidogrel in the Intermediate group than in the other two groups. In comparison with the other two groups, the Long group demonstrated a higher prevalence of diabetes and a higher CCI score. The number of patients with mean hemoglobin < 10 g/dL was 5643 (11.6%), comprising 4319 (11.9%), 1072 (10.2%), and 252 (14.1%) in the Short, Intermediate, and Long groups, respectively (*p* < 0.001).

### 3.2. Hemoglobin Variability According to the Group

**Figure 2:**
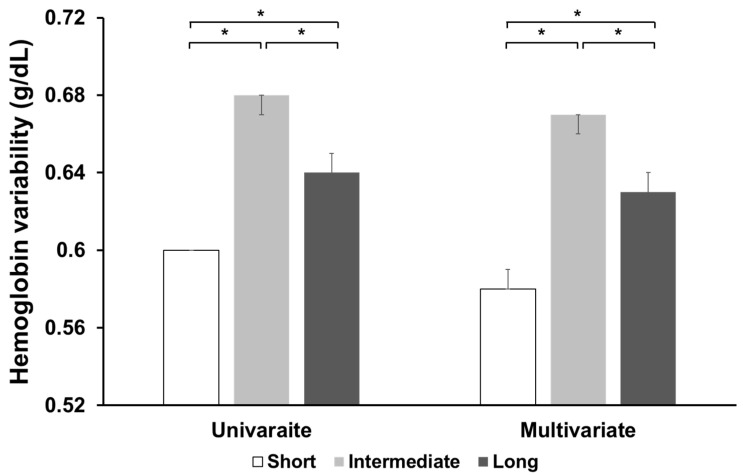
Hemoglobin variability (mean and 95% confidence interval) based on the type of erythropoiesis-stimulating agent. Univariate analysis was performed by one-way analysis of variance, followed by Tukey’s post-hoc test. Multivariate analysis was conducted using analysis of covariance, and adjusting for the following covariates: age, sex, body mass index, vascular access type, diabetes, hemodialysis vintage, Charlson Comorbidity Index score, ultrafiltration volume, Kt/V_urea_, hemoglobin level, serum albumin, serum creatinine, serum phosphorus, and serum calcium, use of renin–angiotensin system blockers, statins, clopidogrel, aspirin, or anti-hypertensive drugs, presence of myocardial infarction or congestive heart failure, erythropoiesis-stimulating agent dose, and erythropoietin resistance index. * *p* < 0.001.

The follow-up duration for the Short, Intermediate, and Long groups was 50 ± 21, 49 ± 20, and 50 ± 21 months, respectively. The hemoglobin variability in the total cohort was 0.58 ± 0.28 g/dL. The hemoglobin variability (mean (95% CI)) was 0.60 (0.60–0.60), 0.68 (0.67–0.68), and 0.64 (0.62–0.65) g/dL in the Short, Intermediate, and Long groups, respectively (Figure 2).

In multivariate analysis, the value was 0.58 (0.58–0.59), 0.67 (0.66–0.67), and 0.63 (0.62–0.64) g/dL in the Short, Intermediate, and Long groups, respectively. The hemoglobin variability was the highest in the Intermediate group and the lowest in the Short group among the three groups.

Multivariate linear regression analysis showed that female sex, diabetes, arteriovenous graft, use of RASBs, aspirin, or anti-hypertensive drugs, and an increase in Kt/V_urea_, mean hemoglobin, serum creatinine, CCI score, or ESA dose were positively associated with hemoglobin variability (Table 2).

However, an increase in age, HD vintage, or body mass index was inversely associated with hemoglobin variability.

We conducted subgroup analyses based on sex, age, CCI, HD vintage, and ESA dose (Table 3).

Overall, the Short group exhibited the lowest hemoglobin variability, whereas the Intermediate group showed the highest hemoglobin variability across the subgroups.

### 3.3. Hemoglobin Variability and Clinical Outcomes

We evaluated all-cause mortality and CVE according to hemoglobin variability. The number of deaths at the end of the follow-up in the Short, Intermediate, and Long groups was 11,472 (31.5%), 3234 (30.8%), and 583 (32.5%), respectively (*p* = 0.949). The number of CVE during the follow-up in the Short, Intermediate, and Long groups was 4113 (16.8%), 1066 (15.5%), and 208 (18.0%), respectively (*p* = 0.023).

**Figure 3:**
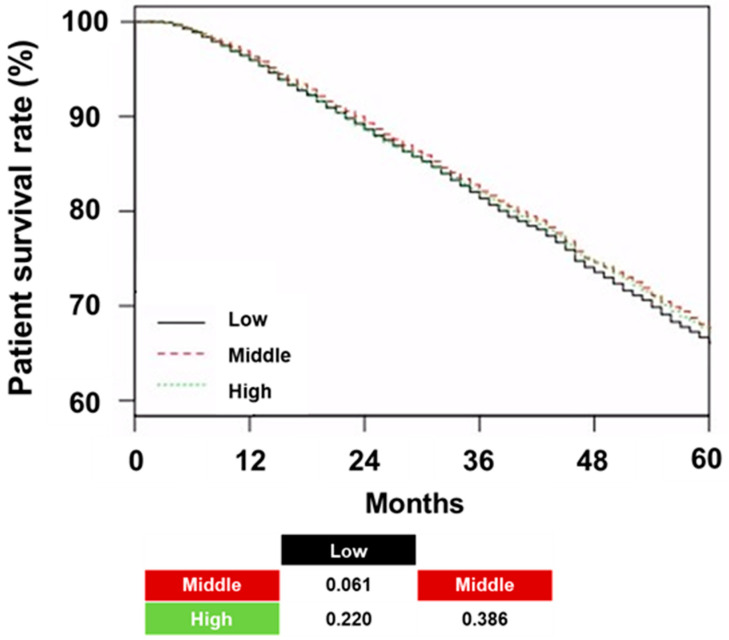
Kaplan–Meier curves stratified by tertiles of hemoglobin variability. The table below the figure presents *p*-values for the survival comparisons between the three groups. Abbreviations: Low, low tertile of hemoglobin variability; Middle, middle tertile of hemoglobin variability; High, high tertile of hemoglobin variability.

The patient survival rates in the low, middle, and high tertiles were 66.1%, 67.7%, and 67.3%, respectively (*p* = 0.060; Figure 3).

No significant differences in patient survival were observed across the tertiles. Neither the trend analysis nor the pairwise comparisons revealed any statistically significant differences. Cox regression did not show a significant association between an increase in hemoglobin variability and all-cause mortality or CVE in univariate and multivariate analyses (Table 4).

In addition, the type of ESA did not show a significant association with all-cause mortality and CVE, except for all-cause mortality in the Long group compared with the Short group.

## 4. Discussion

In this study involving 48,726 HD patients prescribed ESAs, the use of short-acting ESAs was associated with lower hemoglobin variability; however, marginal survival benefit was observed using long-acting ESAs compared with short-acting ESAs. The use of short-acting ESAs decreased hemoglobin variability by approximately 11.8% in the Intermediate group and 6.3% in the Long group. Subgroup analysis revealed that the hemoglobin variability was lower in Short group than in the other two groups.

In our cohort, 74.7% of patients used short-acting ESAs, which highlights the unique circumstances regarding ESA usage and hemoglobin levels in South Korea. Due to insurance policies, ESA administration is restricted if the level of hemoglobin exceeds 11 g/dL. Conversely, if the level of hemoglobin drops below 10 g/dL, the grade of facilities in the HD quality assessment program is negatively affected, ultimately affecting reimbursement. As a result, facilities are under strong pressure to avoid low hemoglobin levels while ensuring ESA use is restricted at high hemoglobin levels, creating a distinctive context. For long- or intermediate-acting agents, ESA administration is halted when the level of hemoglobin exceeds 11 g/dL. Prolonged cessation due to a long half-life often leads to more severe hemoglobin drops, and a subsequent high ESA dose can cause a rapid increase in hemoglobin, contributing to greater hemoglobin variability in patients using intermediate- or long-acting ESAs. In addition, patients using the intermediate/long-acting ESAs tended to have a lower ERI, suggesting that the increase in hemoglobin following ESA administration might have been rapid. In contrast, the use of short-acting ESAs, combined with frequent laboratory follow-ups and dose escalations, likely allowed most patients to maintain hemoglobin levels around 10–11 g/dL. The findings suggest that the administration of short-acting ESAs with regular laboratory monitoring and timely dose adjustments could effectively minimize hemoglobin variability.

Although these practices may have been applied across all three groups in our study, it is hypothesized that short-acting ESAs may be more effective in maintaining low hemoglobin variability due to frequent adjustments based on laboratory results. However, our study has limitations in conclusively demonstrating the effect of ESA properties on hemoglobin variability as an independent factor. Nevertheless, with real-world data, our findings could help predict hemoglobin variability associated with ESA selection in contexts similar to that in South Korea. To clarify the independent effects of different ESA types on hemoglobin variability, a randomized trial excluding the unique circumstances of South Korea would be necessary.

In our study, short-acting ESA use demonstrated the lowest hemoglobin variability, whereas intermediate-acting ESA use showed the highest hemoglobin variability. However, previous studies have reported inconsistent results on hemoglobin variability according to ESA type. A study demonstrated that when medications with the same half-life were administered at longer intervals, hemoglobin variability was decreased [4]. Bernieh et al. compared hemoglobin variability between short- and intermediate-acting ESAs, reporting a trend toward lower hemoglobin variability when using intermediate-acting ESAs compared with short-acting ESAs [5]. In contrast, an analysis utilizing data from the Australian Renal Anemia Group found that hemoglobin variability was higher with intermediate-acting ESAs than with short-acting ESAs [6]. Intermediate- and long-acting ESAs were compared in a study involving pre-dialysis patients, which observed a trend toward lower hemoglobin variability with long-acting ESAs [7]. However, the ARCTOS extension study reported no significant difference in hemoglobin variability between intermediate- and long-acting ESAs [8]. Additionally, a study comparing short- and long-acting ESAs reported a trend toward reduced hemoglobin excursion with long-acting ESAs; however, this difference was not statistically significant [9]. These findings suggest that although longer injection intervals generally reduce hemoglobin variability, factors such as patient characteristics, reimbursement policies, center practices, and patient preferences may contribute to variability. In our study, the need to maintain stable hemoglobin levels, discontinuation of ESA at higher hemoglobin levels, and frequent hemoglobin monitoring may have collectively contributed to the observed lower hemoglobin variability in the Short group.

Our study did not observe a significant association between hemoglobin variability and clinical outcomes. Conventionally, hemoglobin variability has been considered as a risk factor of mortality; however, real-world clinical data have shown inconsistent results, particularly depending on whether non-ESA users were included. Yang et al. analyzed HD patients regardless of ESA use, defining hemoglobin variability with the residual standard deviation as used in our study [13]. Their study reported a hemoglobin variability of 0.60 ± 0.33 g/dL and found that increased hemoglobin variability was associated with higher patient mortality. In contrast, Eckardt et al. included only ESA users, reporting a slightly higher hemoglobin variability of 0.68 ± 0.42 g/dL but failing to demonstrate a relationship between hemoglobin variability and all-cause mortality when measured using the same criteria [19]. In addition, a recent meta-analysis of three studies suggested an association between hemoglobin variability and all-cause mortality; however, the individual studies presented inconsistent results [20]. In comparison with cohorts with only ESA users, inclusion of non-ESA users tends to result in lower overall hemoglobin variability. This also introduces large differences in average hemoglobin levels between ESA users and non-users given that non-users often have lower hemoglobin variability and higher hemoglobin levels, which are associated with better survival. Consequently, the ability to isolate the independent effect of hemoglobin variability is limited.

While hemoglobin variability was extensively examined in this study, our findings showed no significant association between hemoglobin variability and clinical outcomes, such as all-cause mortality or CVE. This suggests that hemoglobin variability alone may not serve as a strong predictor of adverse outcomes. In our cohort of ESA users, the mean hemoglobin variability was 0.58 ± 0.28 g/dL, and changes in hemoglobin variability were not associated with patient survival or CVE. In this study, the relatively short follow-up period of approximately 4 years may have resulted in a lower number of clinical events, potentially affecting the observed outcomes. These findings are consistent with those of Eckardt et al., who similarly included only patients receiving ESAs [19]. Owing to the inconsistent results regarding hemoglobin variability, recent studies have focused on alternative indicators, such as the duration of low hemoglobin levels, hemoglobin amplitude, absolute hemoglobin values, frequency of achieving target ranges, and cumulative ESA dose, as potentially more reliable predictors of clinical outcomes [21,22]. These parameters may more accurately reflect the quality of anemia management and patient prognosis. These indicators could offer greater clinical value in predicting outcomes and informing therapeutic strategies. Future studies are necessary to compare the predictive utility of hemoglobin variability with these alternative indicators in real-world clinical settings.

An analysis of the factors associated with hemoglobin variability reveals that hemoglobin variability was positively associated with female sex, diabetes, arteriovenous graft, use of RASBs, aspirin, or anti-hypertensive drugs, Kt/V_urea_, mean hemoglobin, and serum creatinine. Conversely, it was inversely associated with HD vintage, age, and body mass index. Previous studies have identified factors such as sex, inflammatory status, comorbidities, and RASB usage as determinants of hemoglobin variability, which is consistent with the trends observed in our study [1].

Our study has some limitations. First, it was retrospective, and the baseline characteristics and group sizes differed significantly among the three groups. However, we tried to address these differences through multivariable and subgroup analyses. Second, some important data such as iron status or treatment, injection route of ESAs (subcutaneous or intravenous injection), or pattern or interval of ESA prescriptions were unavailable. These data are crucial for accurately determining hemoglobin levels based on ESA prescriptions. Furthermore, standardization of hemoglobin was not performed due to the web-based collection of relevant data. The lack of validation data and detailed methodology for hemoglobin measurement represents an important limitation of this study. In each HD quality assessment program, participating centers are required to report patient hemoglobin values collected over a 6-month period via a designated online platform. However, details regarding the specific measurement methods are not required. An exception applies to hemoglobin values, which must be reported using measurements obtained through the spectrophotometry method. Therefore, hemoglobin values measured via spectrophotometry were exclusively utilized in this study. However, differences in instrumentation, validation procedures, and potential measurement errors across centers remain inherent limitations that could not be fully addressed within the scope of this study. Despite these limitations, the hemoglobin variability analyzed in this study was based on values collected over a 6-month period in the same patient at the same center, potentially contributing to inter-institutional variability and ensuring internal consistency in individual measurements. However, for other laboratory parameters, information regarding measurement methods, equipment, potential errors, and validation procedures was not available. This represents a significant limitation in this study and in many nationwide studies. Therefore, findings from studies with designs similar to ours should not be considered conclusive. Rather, they should be interpreted as preliminary, highlighting the need for further validation through well-designed prospective studies. Third, our study has limitation for generalization of our results. Studies report inconsistent findings regarding the association between ESA type and hemoglobin variability. These discrepancies may be attributed to differences in study design, patient characteristics, ESA responsiveness, and regional clinical practices, which are influenced by variations in healthcare systems (Table S1) [23,24]. In this study, short-acting ESAs were associated with the lowest hemoglobin variability among the three types evaluated. However, given the influence of multiple confounding factors and the variability observed across previous studies, we acknowledge that our findings may not be fully generalizable to all clinical settings. Therefore, we emphasize the relevance of our results, particularly for regions with healthcare systems and clinical practices similar to those in South Korea. In South Korea, maintaining hemoglobin levels within a narrow target range is strongly emphasized, supported by frequent laboratory follow-up and timely dose adjustments. These practice patterns potentially contribute to the outcomes observed in this study.

## 5. Conclusions

This cohort study reveals that short-acting ESAs are associated with the lowest hemoglobin variability, while intermediate-acting ESAs are associated with the highest variability. In the context of the South Korea healthcare system, which emphasizes frequent hemoglobin monitoring and strict target ranges, the use of short-acting ESAs combined with regular laboratory follow-up appears to support more stable hemoglobin levels. However, these findings should be interpreted with caution due to the observational nature of the study and the absence of direct evidence linking reduced variability to improved clinical outcomes. Furthermore, the results may not be generalizable to healthcare systems with different monitoring practices, reimbursement structures, or treatment protocols. Prospective, multicenter studies are necessary to validate these findings and evaluate their broader applicability.

## Figures and Tables

**Table 1:** Baseline characteristics.

	Short (*n* = 36,420)	Intermediate (*n* = 10,514)	Long (*n* = 1792)	*p*-Value
Age (years)	61.7 ± 12.9	62.7 ± 12.8 ^a^	63.3 ± 12.7 ^a^	<0.001
Sex (male, %)	22,086 (60.6)	5982 (56.9)	1098 (61.3)	<0.001
Hemodialysis vintage (months)	67 ± 68	62 ± 62 ^a^	62 ± 61 ^a^	<0.001
Body mass index (kg/m^2^)	22.7 ± 3.6	22.8 ± 3.6	22.9 ± 3.8 ^a^	0.002
Diabetes (%)	15,914 (43.7)	4743 (45.1)	825 (46.0)	0.009
CCI score	8.8 ± 2.8	8.9 ± 2.9	9.1 ± 3.0 ^ab^	<0.001
Arteriovenous fistula (%)	31,397 (86.2)	9076 (86.3)	1510 (84.3)	0.058
Kt/V_urea_	1.56 ± 0.27	1.61 ± 0.29 ^a^	1.61 ± 0.28 ^a^	<0.001
Ultrafiltration volume (L/session)	2.3 ± 0.9	2.2 ± 0.9 ^a^	2.2 ± 0.9 ^a^	<0.001
Hemoglobin (g/dL)	10.6 ± 0.6	10.7 ± 0.6 ^a^	10.6 ± 0.6 ^b^	<0.001
Serum albumin (g/dL)	4.03 ± 0.33	3.96 ± 0.33 ^a^	3.93 ± 0.31 ^ab^	<0.001
Serum phosphorus (mg/dL)	5.0 ± 1.2	4.8 ± 1.2 ^a^	4.8 ± 1.2 ^a^	<0.001
Serum calcium (mg/dL)	8.9 ± 0.7	8.8 ± 0.7 ^a^	8.8 ± 0.7 ^a^	<0.001
Serum creatinine (mg/dL)	9.6 ± 2.6	9.4 ± 2.6 ^a^	9.2 ± 2.6 ^ab^	<0.001
Use of RASBs (%)	24,193 (66.4)	7359 (70.0)	1238 (69.1)	<0.001
Use of aspirin (%)	18,073 (49.6)	4756 (45.2)	897 (50.1)	<0.001
Use of clopidogrel (%)	9652 (26.5)	2542 (24.2)	481 (26.8)	<0.001
Use of statins (%)	17,920 (49.2)	5572 (53.0)	932 (52.0)	<0.001
Use of anti-hypertensive drugs (%)	31,144 (85.5)	9080 (86.4)	1538 (85.8)	0.090
MI or CHF	21,002 (57.7)	6417 (61.0)	1109 (61.9)	<0.001
ESA dose (IU/week)	8119 ± 4498	6035 ± 4119 ^a^	4116 ± 2374 ^ab^	<0.001
ERI [(IU/week)/kg/(g/dL)]	13.3 ± 8.1	10.0 ± 7.9 ^a^	6.7 ± 4.5 ^ab^	<0.001
Follow-up duration (months)	50 ± 21	49 ± 20 ^a^	50 ± 21 ^b^	<0.001

Data are expressed as means ± standard deviations for continuous variables and as numbers (percentages) for categorical variables. The *p*-values were tested by one-way analysis of variance, followed by Tukey’s post-hoc test. Pearson’s χ^2^ test was performed for categorical variables: ^a^ *p* < 0.05 vs. Short group; ^b^ *p* < 0.05 vs. Intermediate group. Abbreviations: CCI, Charlson Comorbidity Index; CHF, congestive heart failure; ERI, erythropoietin resistance index; ESA, erythropoiesis-stimulating agent; IU, international unit; Kt/V_urea_, hemodialysis adequacy calculated using Daugirdas equation; MI, myocardial infarction; RASB, renin–angiotensin system blocker.

**Table 2:** Regression analysis for variables associated with hemoglobin variability.

	Univariate		Multivariate	
	Estimate (SE)	*p*-Value	Estimate (SE)	*p*-Value
Group (ref: Short group)				
Intermediate group	0.080 (0.003)	<0.001	0.081 (0.003)	<0.001
Long group	0.036 (0.007)	<0.001	0.046 (0.007)	<0.001
Age (per 1 year)	−0.001 (0.000)	<0.001	−0.001 (0.000)	<0.001
Sex (ref: males)	0.030 (0.003)	<0.001	0.031 (0.004)	<0.001
HD vintage (per 10 months)	0.002 (0.000)	<0.001	−0.003 (0.000)	<0.001
BMI (per 1 kg/m^2^)	−0.009 (0.000)	<0.001	−0.010 (0.001)	<0.001
Diabetes (ref: non-diabetes)	0.012 (0.003)	<0.001	0.016 (0.003)	<0.001
CCI score (per 1 score)	0.001 (0.000)	0.041	0.002 (0.001)	0.007
Vascular access (ref: AVF)	0.017 (0.004)	<0.001	0.011 (0.003)	0.006
Kt/Vurea	0.077 (0.005)	<0.001	0.024 (0.006)	<0.001
UFV (per 1 L/session)	−0.007 (0.001)	<0.001	−0.002 (0.002)	0.275
Mean hemoglobin (per 1 g/dL)	0.016 (0.002)	<0.001	0.034 (0.003)	<0.001
Serum albumin (per 1 g/dL)	−0.001 (0.004)	0.844	−0.001 (0.005)	0.744
Serum phosphorus (per 1 mg/dL)	−0.002 (0.001)	0.113	−0.002 (0.001)	0.126
Serum calcium (per 1 mg/dL)	−0.002 (0.002)	0.253	−0.000 (0.002)	0.914
Serum creatinine (per 1 mg/dL)	−0.000 (0.000)	0.548	0.004 (0.001)	<0.001
Use of RASBs	0.054 (0.003)	<0.001	0.035 (0.004)	<0.001
Use of aspirin	0.007 (0.003)	0.004	0.018 (0.003)	<0.001
Use of clopidogrel	−0.006 (0.003)	0.027	−0.002 (0.003)	0.516
Use of statins	−0.002 (0.003)	0.532	−0.003 (0.003)	0.368
Use of anti-hypertensive drugs	0.060 (0.004)	<0.001	0.027 (0.005)	<0.001
MI or CHF	0.004 (0.003)	0.090	−0.003 (0.003)	0.285
ESA dose (per 1000 IU/week)	0.001 (0.000)	<0.001	0.003 (0.001)	0.015
ERI (per 10 (IU/week)/kg/(g/dL))	0.017 (0.002)	<0.001	0.008 (0.007)	0.264

Multivariate analysis was adjusted for age, sex, body mass index, vascular access type, diabetes, hemodialysis vintage, Charlson Comorbidity Index score, ultrafiltration volume, Kt/V_urea_, levels of hemoglobin, serum albumin, serum creatinine, serum phosphorus, and serum calcium, use of renin–angiotensin system blockers, statins, clopidogrel, or aspirin, presence of myocardial infarction or congestive heart failure, ESA dose per week, and erythropoietin resistance index. Abbreviations: AVF, arteriovenous fistula; BMI, body mass index; CCI, Charlson Comorbidity Index; CHF, congestive heart failure; HD, hemodialysis; ERI, erythropoietin resistance index; ESA, erythropoiesis-stimulating agent; IU, international unit; Kt/V_urea_, hemodialysis adequacy calculated using Daugirdas equation; MI, myocardial infarction; RASB, renin–angiotensin system blocker; ref, reference group; SE, standard error; UFV, ultrafiltration volume.

**Table 3:** Hemoglobin variability according to subgroup.

	Univariate	Multivariate		Univariate	Multivariate
	Mean (95% CI)	Mean (95% CI)		Mean (95% CI)	Mean (95% CI)
Males			Females		
Short	0.59 (0.59–0.59)	0.57 (0.57–0.58)		0.62 (0.61–0.62)	0.60 (0.60–0.61)
Intermediate	0.67 (0.66–0.67) ^a^	0.65 (0.64–0.66) ^a^		0.70 (0.69–0.71) ^a^	0.69 (0.68–0.70) ^a^
Long	0.62 (0.61–0.64) ^ab^	0.62 (0.60–0.63) ^ab^		0.66 (0.64–0.68) ^ab^	0.65 (0.63–0.67) ^a^
<65 years old			≥65 years old		
Short	0.61 (0.60–0.61)	0.59 (0.59–0.60)		0.59 (0.59–0.60)	0.57 (0.57–0.58)
Intermediate	0.68 (0.68–0.69) ^a^	0.67 (0.66–0.68) ^a^		0.67 (0.67–0.68) ^a^	0.66 (0.65–0.67) ^a^
Long	0.64 (0.63–0.66) ^ab^	0.64 (0.62–0.66) ^a^		0.63 (0.61–0.65) ^ab^	0.62 (0.60–0.64) ^ab^
CCI < 9			CCI ≥ 9		
Short	0.60 (0.59–0.60)	0.58 (0.58–0.59)		0.60 (0.60–0.61)	0.59 (0.58–0.59)
Intermediate	0.69 (0.68–0.69) ^a^	0.67 (0.66–0.68) ^a^		0.67 (0.67–0.68) ^a^	0.66 (0.65–0.67) ^a^
Long	0.63 (0.61–0.65) ^ab^	0.63 (0.61–0.65) ^ab^		0.64 (0.62–0.65) ^ab^	0.63 (0.61–0.65) ^ab^
HDV < 40 mo			HDV ≥ 40 mo		
Short	0.60 (0.60–0.61)	0.59 (0.59–0.60)		0.59 (0.59–0.60)	0.57 (0.57–0.58)
Intermediate	0.69 (0.68–0.69) ^a^	0.68 (0.67–0.69) ^a^		0.67 (0.66–0.68) ^a^	0.65 (0.64–0.66) ^a^
Long	0.66 (0.64–0.67) ^ab^	0.65 (0.63–0.67) ^ab^		0.62 (0.60–0.63) ^b^	0.61 (0.59–0.63) ^ab^
Low ESA dose			High ESA dose		
Short	0.58 (0.58–0.59)	0.57 (0.57–0.58)		0.61 (0.61–0.62)	0.60 (0.59–0.60)
Intermediate	0.66 (0.65–0.66) ^a^	0.64 (0.63–0.64) ^a^		0.73 (0.72–0.74) ^a^	0.72 (0.71–0.73) ^a^
Long	0.63 (0.62–0.65) ^ab^	0.61 (0.60–0.63) ^a^		0.67 (0.63–0.71) ^b^	0.67 (0.63–0.71) ^a^

Multivariate analysis was adjusted for age, sex, body mass index, vascular access type, diabetes, hemodialysis vintage, CCI, ultrafiltration volume, Kt/V_urea_, levels of hemoglobin, serum albumin, serum creatinine, serum phosphorus, and serum calcium, use of renin–angiotensin system blockers, statins, clopidogrel, aspirin, or anti-hypertensive drugs, presence of myocardial infarction or congestive heart failure, ESA dose, and erythropoietin resistance index. ^a^ *p* < 0.05 vs. Short group; ^b^ *p* < 0.05 vs. Intermediate group. Abbreviations: CCI, Charlson Comorbidity Index; CI, confidence interval; HDV < 40 mo, hemodialysis vintage of less than 40 months; HDV ≥ 40 mo, hemodialysis vintage of 40 months or more longer; ESA, erythropoiesis-stimulating agent; Low ESA dose, subgroup with ESA dose < 6960 IU/week; High ESA dose, subgroup with ESA dose ≥ 6960 IU/week.

**Table 4:** Hemoglobin variability and clinical outcomes.

	Univariate		Multivariate	
	HR (95% CI)	*p*	HR (95% CI)	*p*
All-cause mortality				
HBV (per 1 g/dL)	0.98 (0.93–1.04)	0.556	1.04 (0.98–1.10)	0.170
Intermediate group (ref: Short)	1.00 (0.96–1.04)	0.872	0.99 (0.95–1.03)	0.699
Long group (ref: Short)	1.02 (0.94–1.11)	0.660	0.91 (0.84–0.99)	0.031
Long group (ref: Intermediate)	1.02 (0.93–1.11)	0.873	0.92 (0.84–1.10)	0.059
CVE				
HBV (per 1 g/dL)	0.92 (0.84–1.02)	0.097	0.95 (0.86–1.05)	0.357
Intermediate group (ref: Short)	0.94 (0.87–1.00)	0.052	0.95 (0.88–1.01)	0.121
Long group (ref: Short)	1.08 (0.94–1.24)	0.300	1.02 (0.88–1.17)	0.834
Long group (ref: Intermediate)	1.15 (0.99–1.34)	0.064	1.07 (0.92–1.25)	0.356

Multivariate analysis was adjusted for age, sex, body mass index, vascular access type, diabetes, hemodialysis vintage, Charlson Comorbidity Index score, ultrafiltration volume, Kt/V_urea_, levels of hemoglobin, serum albumin, serum creatinine, serum phosphorus, and serum calcium, use of renin–angiotensin system blockers, statins, clopidogrel, aspirin, or anti-hypertensive drugs, presence of myocardial infarction or congestive heart failure, erythropoiesis-stimulating agent dose, and erythropoietin resistance index. Abbreviations: CI, confidence interval; CVE, cardiovascular events; HBV, hemoglobin variability; HR, hazard ratio; ref, reference group.

## Data Availability

Raw data were generated at the Health Insurance Review and Assessment Service. The database can be requested from the Health Insurance Review and Assessment Service by sending a study proposal, including the purpose of the study, study design, and duration of analysis, on the web site (https://www.hira.or.kr (accessed on 8 July 2025)). The authors cannot distribute the data without permission.

## Supplementary Materials

The following supporting information can be downloaded at https://www.mdpi.com/article/10.3390/jcm14144863/s1, Table S1. Factors influencing the selection of ESA.
