# Supplementary material for: Effect of Erythropoiesis-Stimulating Agent Types on Hemoglobin Variability in Hemodialysis Patients
Source: J Clin Med. 2025 Jul 9;14(14):4863. doi: 10.3390/jcm14144863 (PMC12295620; doi:10.3390/jcm14144863)
Supplement: Supplementary file 1 [file jcm-14-04863-s001.zip › jcm-3678678-supplementary.pdf]

Table S1. Factors influencing the selection of ESA

Level	Factors influencing ESA selection
**Patient level**	● ESA efficacy or patient responsiveness<br>● Cost<br>● Patient preference<br>● Safety profile<br>● Severity of anemia<br>● Dialysis vintage<br>● Treatment adherence<br>● Comorbid conditions
**Facility level**	● Medical staff workload or preference<br>● ESA availability or storage capacity<br>● Facility-level cost considerations
**Healthcare system level**	● Reimbursement policies<br>● Insurance coverage<br>● National or regional availability
	● Clinical policies or prescribing guidelines (e.g., target hemoglobin range)

Abbreviation: ESA, erythropoiesis-stimulating agent
